# Notices

**Published:** 1867-08

**Authors:** 


### NOTICES.

DICKENS' Works, in seven volumes, bound uniformly in black cloth, by Peterson Brothers of Philadelphia, for Twenty Dollars. If any readers of the JOURNAL will send us twenty names of new subscribers to Hall's Journal of Health for one year, with thirty dollars, the publisher will deliver to their order one set of Dickens' Works for each twenty subscriptions so sent in.

Waverly Novels complete, five volumes, $25, will be furnished for twenty new names and $18.

A FAMILY NEWSPAPER. — We have frequently drawn the attention of our readers to the SCIENTIFIC AMERICAN, published weekly at No. 37 Park Row, New York, for $3 a year. It does not discuss politics or religion but is always on the side of good morals, the diffusion of intelligence and the encouragement of good citizanship. It is a reliable publication; it does not advocate one thing to-day and something different to-morrow; it takes up no opinion hastily, pins its faith to no great name, but in the truth; it investigates before it announces; it takes nothing for granted even if wise men say it; and although it discusses scientific questions in the mechanic arts, yet it is so eminently practical that every housekeeper will be instructed in domestic matters in every issue for it gives the earliest information as to all improvements in every department of human life. Often in a single number there is practical information as to the conduct of life worth intrinsically more than a year's subscription. Taking up an issue at random we find in part the following subjects: How to buy meat; how to preserve animal substances; beef cured by venous injection; new air pump; a new gas engine; value of different kinds of fuel; breaking of lamp chimnies; fire-proof coating for floors, &c., &c.

If we were asked which was one of the very best religious newspapers we should answer, "The Boston Christian Watchman & Reflector." Another well edited paper for religious family reading, we do not remember now of what denomination, is THE TELESCOPE, published at Dayton, Ohio. But a great defect of most of the religious newspapers is their barrenness of editorials pertaining to the times. The remedy is to pay editors larger salaries so that they may have nothing else to do but edit the paper and write for it; greater salaries will command greater minds; every church member has a duty to perform in this regard, not only to take a religious newspaper himself but to use his personal effort and influence and persuasion to induce his friends and neighbors to take one. No family in the United States ought to be without a weekly religious newspaper, because it is a power for good in the land, and is needed, greatly needed — more and more needed every day to antagonize the covert attacks which the secular and all the "Sunday" papers are constantly making against the Sabbath, against the Bible, against the clergy and against professing christians in general; and to antagonize, too, the trash reading which at last has become part and parcel of most of our city dailies. These things imperatively demand the attention of all thoughtful men.

Annihilation. — The American Tract Society, at 28 Cornhill, Boston, and 13 Bible House, New York, have issued a twenty-five cent book entitled "The Wicked not Annihilated," being a refutation of Modern Sadduceism, by Rev. Israel P. Warren. Also a beautiful volume of 262 pps, entitled "Friendly Words with Fellow Pilgrims," by James William Kimball. Among the subjects are: Waiting for Deep Impressions; I have no Faith; Assurance; Every Christian a Worker; How to save Souls; Your Mission. — 50 cents. Also a beautiful 12mo of 358 pps., white paper and clear type, $1 25, entitled

"GOD'S WORD WRITTEN,"

explaining and proving the doctrine of the inspiration of the Holy Scriptures, by the Rev Edward Garbett, M, A., incumbent of Christ Church, Surbitan and select preacher of the University of Oxford, England.

Thoughtful and observant men know that there is a growing feeling and disposition to call in question the Divine origin of Scripture truth; a wish, as it were, that the Bible should not be the word of God, and this in the face of the patent fact that it opposes all that is evil and espouses all that is good. Well has it been said that good men or angels could not have written it because in saying that it is the "Word of God" they would have told a falsehood. Devils and evil spirits would not have written such a book, because it everyw here condems them, shows their wiles their deceits and their wickedness, and condems them to an existance of unutterable woe, and as there is no other class of intellectual beings in the universe God only could have been the author of such a book. — Among the contents are. "What is Christianity?;" Christianity identified with the Bible; Authority of the Scriptures; The whole Scriptures are the Word of God; The Word of God is verbally inspired. — Nineteen chapters in all. This book will greatly confirm the true believer; it will establish the doubting and will lead the thoughtless to reflect and build on the sure foundation-stone. Mother, get your young daughter to read it; father put it in the way of your son — it may be salvation to him. It is a good book for everybody, young and old, saint and sinner, for it is "God's Word Written."

### *Notices.*

THE HOME JOURNAL, $3 a year, is published, as formerly, at 107 Fulton street, and is so enlarged and beautified as to be scarcely recognized as an old acquaintance. Morris and Willis are no more in it, nor will their equals in their line ever be; they were the Home Journal personified in it, but they have left us for aye and their successors have laid themselves out for the work of making one of the most inviting of weekly publications and they have gathered around them an array of talent which will doubtless give it a position and a circulation which it has never yet attained. Morris, Phillips & Co., Publishers.

REPORT of the Pennsylvania Hospital for the Insane, for 1866, by Thomas M. Kirkbride, M. D., Physician-in-Chief and Superintendent,—published by order of the Board of Managers. Intemperance as a cause of insanity is brought forcibly to notice in the statement that "Much of the ill health, the loss of property, domestic difficulties, disappointed expectations and mental anxiety, in not a few instances, were the consequences of intemperance on the part of parents, husbands, or the members of families, and without which the disease would not have been developed." — Among the noteworthy improvements are the introduction of gymnasiums, flower gardens, reading-rooms and evening entertainments as the result of Dr. Kirkbride's wisdom, thoughtfulness and perseverence; and what a change from the pens and iron cages and chains to which the helpless insane were doomed but a few short years ago, and in some places still, which that angel of mercy and kindness, Dorothea Dix, has yet not been able to visit.

CRAZY FARMERS. — Right soundly were we handled a few years ago for making the statement that there were more crazy farmers than of any other class, and in this Report it is verified that of nearly five thousand patients there were one-third more farmers than of any other class sent to them; more merchants and clerks next — what a graceless occupation is that of a clerk, a drudge at best; what temptation for ill requited labor, and above all the discouraging consideration that if he barely discharges his duties he gets his full pay, but whatever of fidelity and of extra efforts to forward the interests of his employers he gets no more than his full pay; and then for a young man to place himself voluntarily under the whim and caprice of his employer, often an ignorant man, too often an unprincipled one, is a degrading thought; better a thousand times learn some handicraft and being complete master of your trade make your employer bow to you, young man, for who does not know that in the city of New York an accomplished workman can command his own terms and can select his own patrons. "He's nothing but a clerk," is the contemptous expression on a thousand lips. Where is your manhood, young gentlemen?

### *Notices.*

Two hundred "clerks" in one insane asylum! and only fifty doctors; these hav'nt time to go crazy; plenty of practice, good fees and the pets of all the women—for where is the woman who does not believe in her doctor! Authors — One!! The reason of that is their insanity runs out at their finger's ends, they diffuse it among the people, vent is given to thought and all that is left of him is milk-and-water — a perfectly harmless compound; as proof, let any reader go and talk half an hour with any author, and if you do not get sick and tired of him at the "end of the first quarter" then we are mistaken. Only one crazy author out of five thousand insane! Reader, if you have fears at any time of going crazy, just go and write a book

To C. O. H. — Can't afford to read a three-page letter from any correspondent, but gather the main drift, perhaps, from a few first lines; we can not tell all about any one thing in one article; it would be too long; nobody would read it; we have written a whole book about Sleep; if you write again and want to be read, say all you want on one peice of common note-paper; enough can be said in that space to last a year. Ye long winded folk: your blows are not worth a button; come to your subject at once; if two words express your meaning, select the one having the fewest syllables; if two monosyllables will equally convey your idea write the one which has the fewest letters. Just imagine that every letter you want printed cost half a dime, and what wordy fellow has many!—and act accordingly.

Persons often send a dollar or two for the Journal or for a book, and then write a letter a mile long, detailing their signs and symptoms, with insufferable diffuseness, and seem to think that an opinion or a prescription will be thrown in; when the editor opens a letter longer than a few words and in a strange hand, and has no money in it — it is turned over to another to glean the one main idea and report it. Time is money in a large city A whole bundle of compliments would not buy a sprig of parsley for a bowl of soup. If you make a purchase at a store you do not expect to have some other thing of equal or greater cost thrown in, because you have patronized the "House." It would take us a year to answer all the letters we receive in a week from persons who seem to think that their subscribing for the Journal, or purchasing a book or speaking praises, entitles them to a prescription. Whoever wants a letter from us on any subject, must send with it Five Dollars.

